# Polymorphisms in the genes encoding RLR and TLR3 and CMV DNAemia in subjects coinfected with human immunodeficiency virus and cytomegalovirus

**DOI:** 10.1007/s00705-024-06114-3

**Published:** 2024-09-27

**Authors:** Agnieszka Jabłońska, Elżbieta Jabłonowska, Mirosława Studzińska, Juliusz Kamerys, Edyta Paradowska

**Affiliations:** 1 Laboratory of Virology, Institute of Medical Biology, Polish Academy of Sciences, 106 Lodowa St, Lodz, 93-232 Poland; 2 Department of Infectious Diseases and Hepatology, Medical University of Lodz, Lodz, Poland

**Keywords:** Cytomegalovirus, Human immunodeficiency virus, Pattern-recognition receptors, Single-nucleotide polymorphism

## Abstract

Cytomegalovirus (CMV) is a pathogen that is common worldwide and is often present in individuals infected with human immunodeficiency virus (HIV). Pattern recognition receptors (PRRs) are host sensors that activate the immune response against infectious agents. However, it is unclear whether PRR single-nucleotide polymorphisms (SNPs) are associated with the occurrence of CMV DNAemia in subjects coinfected with HIV and CMV. HIV/CMV-coinfected patients with and without CMV DNAemia were recruited for this study. The *DDX58* rs10813831 and *IFIH1* (rs3747517 and rs1990760) polymorphisms were genotyped using the TaqMan Allelic Discrimination Assay, whereas the *DDX58* rs12006123 and *TLR3* (rs3775291 and rs3775296) SNPs were analyzed using a polymerase chain reaction restriction fragment length polymorphism (PCR-RFLP) assay. A mutation present in at least one allele of the *DDX58* rs12006123 SNP occurred at least two times more frequently in HIV/CMV-coinfected patients with CMV DNAemia than in coinfected subjects without CMV DNAemia (OR, 2.50; 95% CI, 1.33–4.68; *p* = 0.004, in the dominant model). A higher level of CMV DNAemia was observed in subjects who had the heterozygous (GA) or homozygous recessive (AA) genotype for the *DDX58* rs12006123 SNP compared with those who had the wild-type (GG) genotype (*p* = 0.0003). Moreover, in subjects with a mutation detected in at least one allele of the *DDX58* rs12006123 SNP, a lower serum IFN-β concentration was found compared with those who had a wild-type (GG) genotype for this polymorphism (*p* = 0.024). The *DDX58* rs12006123 SNP is associated with CMV DNAemia in HIV/CMV-coinfected patients.

## Introduction

Human immunodeficiency virus (HIV), a member of the genus *Lentivirus*, family *Retroviridae*, is one of the most common bloodborne pathogens and one of the most devastating human diseases of the 21st century. In HIV-infected individuals, coinfection with cytomegalovirus (CMV; genus *Cytomegalovirus*, family *Orthoherpesviridae*) contributes to increased systemic inflammation, chronic immune activation, and immunosenescence, despite effective antiretroviral therapy (ART) [1, 2]. In HIV-positive patients who are stable on ART, the associations between levels of CMV antibodies, cardiovascular risk, and neurocognitive health are moderated by age-associated increases in the response to CMV, while an independent link between CMV antibodies and insulin resistance has been observed [3]. Additionally, active CMV replication is common in HIV-infected individuals [4, 5] and is associated with low CD4^+^/CD8^+^ ratios [1, 5].

During viral infections, pattern recognition receptors (PRRs), including endosomal Toll-like receptors (TLRs) and cytoplasmic RNA helicases such as retinoic acid-inducible gene (RIG)-I-like receptors (RLRs) initiate antiviral immunity *via* induction of type I and III interferons (IFNs) as well as inflammatory cytokines. These receptors are expressed in various tissues and cell types, including dendritic cells, neutrophils, macrophages, lymphocytes, and epithelial cells [6, 7]. It has been reported that the homozygous recessive (TT) genotype of the *TLR3* rs3775291 single-nucleotide polymorphism (SNP) provided resistance to HIV infection in Spanish HIV-exposed seronegative (HESN) intravenous drug users (IDUs), and individuals with this genotype sustained lower levels of viral replication [8]. In another study, a mutation present in at least one allele of this SNP was also associated with protection against HIV infection in Estonian HESN IDUs [9]. An association between CT and TT genotypes of the *TLR9* 2848C/T SNP and the occurrence of CMV DNAemia in HIV/CMV-coinfected patients has also been described [4]. Here, we report a correlation between *DDX58* rs12006123 SNP and CMV DNAemia in HIV/CMV-coinfected patients.

The aim of this investigation was to assess whether polymorphisms in the TLR3 and RLR genes (*DDX58*, encoding RIG-I and *IFIH1*, encoding MDA5) have an impact on the occurrence of CMV DNAemia in HIV-infected patients with a CMV-seropositive status. The association between these SNPs and the concentrations of selected cytokines was also studied.

## Materials and methods

### Participants

Whole peripheral blood and serum samples were collected from 192 HIV/CMV-coinfected adults (median age, 37.7; range, 17–72 years) recruited from the Department of Infectious Diseases and Hepatology, Medical University of Lodz (Lodz, Poland). Subjects were consecutively enrolled from November 2006 to August 2008 and from May 2017 to March 2018. Patients with positive tests for HIV and CMV antibodies were included in the study. All HIV-infected patients received combined ART (e.g., tenofovir [TDF or TAF]/emtricitabine [FTC], abacavir [ABC]/lamivudine [3TC] combined with an integrase or protease inhibitor or a nonnucleoside reverse transcriptase inhibitor). HIV quantification was performed using the Cobas AmpliPrep/Cobas TaqMan HIV-1 test, v2.0 (Roche Diagnostics GmbH, Mannheim, Germany) on a Light Cycler 480 thermal cycler (Roche Diagnostics GmbH). CMV infection was diagnosed by the detection of CMV DNA in whole blood of patients with specific anti-CMV IgG antibodies present in their serum samples. CMV IgG was measured using LIAISON assay (DiaSorin, Saluggia, Italy) according to the manufacturer’s recommendations. CMV DNAemia was quantified in all participants after study entry at a median of 4.9 years after initiation of ART (range, 0.3–21.4 years; Table 1). All participants included in the study were of Caucasian origin and were recruited from the same geographical region. This study was performed in accordance with the Helsinki Declaration and with good clinical practice guidelines and was approved by the appropriate ethics committees (RNN/211/06/KE and RNN/33/17/KE). All volunteers gave written informed consent to donate samples for research purposes.

**Table 1:** Demographic, clinical, and virological characteristics of the HIV/CMV-coinfected patients

Variable	Patients with CMV DNAemia (*n* = 108)	Patients without CMV DNAemia (*n* = 84)	*p*-value^a^
Age (median and range, years)	35.3 (17-72 years)	40.9 (18-63 years)	< 0.0001
CD4^+^ T cell count, median and range			
• ≤ 200 cells/mm^3^	93.5 (9-200)	148 (7-198)	0.239
• > 200 cells/mm^3^	558 (237-1959)	495 (206-1220)	0.233
CD4^+^ T cell nadir, median and range			
• ≤ 200 cells/mm^3^	72 (1-200)	102 (4-198)	0.103
• > 200 cells/mm^3^	338 (222-659 )	397 (224-624)	0.570
ART time (median and range, years)	3.1 (0.5-12.0)	5.9 (0.3-21.4)	0.003^b^
HBsAg positive, *n* (%)	21/108 (19.4)	9/84 (10.7)	0.112
Anti-HCV positive, *n* (%)	30/108 (27.8)	16/84 (19.5)	0.176
Self-reported HIV-1 transmission route			
• MSM	67/108 (62.0)	39/84 (46.4)	0.040
• HET	16/108 (14.8)	24/84 (28.6)	0.031
• MSM/HET	2/108 (1.9)	2/84 (2.4)	1.000
• IDU	23/108 (21.3)	19/84 (22.6)	0.862
Cytokines, median and range (pg/ml)			
• IFN-α	0.0 (0.0-160.5)	0.0 (0.0-93.6)	0.038
• IFN-β	0.0 (0.0-2935)	0.0 (0.0-2922)	0.051
• IL-4	0.0 (0.0-11.48)	0.0 (0.0-36.79)	0.408
• IL-6	13.80 (9.42-75.99)	6.98 (0.0-12.29)	0.008
• IL-7	75.05 (0.0-820.0)	30.71 (0.0-1100.0)	0.208
• IL-10	30.24 (0.0-64.21)	8.49 (0.0-48.05)	0.166
• TNF-α	32.37 (15.65-56.99)	29.93 (16.78-40.45)	0.536

^a^ Fisher’s exact test (percentages) or Mann-Whitney U test (continuous variables);

^b^ Pearson’s correlation coefficient r = -0.304, *p* = 0.009; *n*, number of subjects (%); ART, antiretroviral therapy; HBsAg hepatitis B virus surface antigen; anti-HCV, antibodies against hepatitis C virus; MSM, men having sex with men; HET, heterosexual transmission; IDU, injecting drug use

### Analysis of RLR and TLR SNPs

Genomic DNA was isolated from peripheral blood using a QIAamp DNA Blood Mini Kit (QIAGEN GmbH, Hilden, Germany) according to the manufacturer’s recommendations. The *DDX58* rs10813831 (C_1552406_10), *IFIH1* rs3747517 (C_25984418_10), and *IFIH1* rs1990760 (C_2780299_30) SNPs were genotyped using a TaqMan Allelic Discrimination Assay (Applied Biosystems, Carlsbad, CA, USA) and TaqMan Genotyping Master Mix (Thermo Fisher Scientific, Vilnius, Lithuania) using a 7900HT Fast Real-Time PCR system (Applied Biosystems, Foster City, CA, USA). SNP genotyping was performed using 10 ng of genomic DNA, 1.25 µl of TaqMan Allelic Discrimination Assay Mix, and 12.50 µl of TaqMan Genotyping Master Mix in a final volume of 25 µl. The initial denaturation step was performed at 95°C for 10 min, followed by 40 cycles of denaturation at 95°C for 15 s and annealing at 60°C for 60 s. Genotyping for the *DDX58* rs12006123 and *TLR3* (rs3775291 and rs3775296) SNPs was performed using a polymerase chain reaction restriction fragment length polymorphism (PCR-RFLP) assay as described elsewhere [10, 11]. In brief, PCR products were digested with the restriction enzyme SsiI (AciI), HpyF3I, or MboII (Fermentas, Hanover, MD, USA), and the digested fragments were separated using a QIAxcel capillary electrophoresis system (QIAGEN GmbH, Hilden, Germany). Randomly selected samples of each PRR SNP were confirmed using a 96-capillary 3730xl DNA Analyzer (Applied Biosystems).

### Assessment of CMV DNAemia

CMV DNAemia was defined as the presence of detectable CMV DNA in the patient’s blood samples. The CMV DNA copy number was determined using a 7900HT Fast Real-Time PCR System (Applied Biosystems) as described previously [12]. The amplification was performed using a primer set recognizing sequences within the *UL55* gene (gB1, GAG GAC AAC GAA ATC CTG TTG GGC A; gB2, GTC GAC GGT GGA GAT ACT GCT GAG G; TaqMan probe, CAA TCA TGC GTT TGA AGA GGT AGT CCA) [12]. Briefly, PCR assays were carried out in a final volume of 25 µl containing 5 µl of patient DNA, 12.5 µl of TaqMan Universal PCR Master Mix (Applied Biosystems), 0.1 µl of the primer set (100 pmol/µl), 0.05 µl of probe (100 pmol/µl) labeled with FAM (6-carboxyfluoroscein) and TAMRA (6-carboxytetramethylrhodamine), and 7.25 µl of deionized water. DNA samples were amplified as follows: preincubation at 95°C for 10 min, followed by 50 cycles of 15 s at 95°C and 60 s at 60°C. To validate the assay, a negative control without template DNA was included in each amplification run. The analytical sensitivity of the assay was determined to be 2 × 10^2^ copies/ml. The *UL55* gene was cloned using a TOPO Kit for Sequencing, with One Shot TOP10 Chemically Competent *E. coli* (Invitrogen, Carlsbad, CA, USA) according to the manufacturer’s instructions. The resulting clones were sequenced in both directions to confirm that no point mutations had been introduced during the amplification process. High-quality plasmid DNA was purified using a PureLink Quick Plasmid Miniprep Kit (Invitrogen) according to the manufacturer’s recommendations.

### Measurement of cytokine levels

Cytokine levels were measured using a BD Cytometric Bead Array (CBA) Human Th1/Th2/Th17 Cytokine Kit (BD Biosciences, San Jose, CA, USA). This assay simultaneously measures the concentration of IL-2, IL-4, IL-6, IL-10, IL-17A, TNF, and IFN-γ. Serum samples were measured on an LSR II BD flow cytometer (BD Biosciences, Franklin Lakes, NJ, USA) and analyzed using FCAP Array software (BD Biosciences). Levels of IFN-α, IFN-β (PBL Assay Science, Piscataway, NJ, USA), and IL-7 (Thermo Scientific, Frederick, MD, USA) were determined using ELISA kits according to the manufacturer’s instructions.

### Statistical analysis

Statistical analysis was performed using GraphPad Prism version 5.0 (GraphPad Software, San Diego, CA, USA) or SPSS 25.0 for Windows (SPSS Inc., Chicago, Illinois, USA). Baseline data were expressed as the median and range for nonnormal continuous variables or numbers (percentages) for categorical variables. Experimental data were analyzed using nonparametric Fisher’s exact test, the chi-square test, or the Mann-Whitney U test, when appropriate. Differences were regarded as statistically significant when the *p*-value was ≤ 0.05. Hardy-Weinberg equilibrium (HWE), linkage disequilibrium (LD), and haplotype analyses were performed using SNPStats software (http://www.snpstats.net/start.htm). The *p*-values were corrected for multiple tests with a Bonferroni correction; the significance level for *p*_*B*_ was 0.010 instead of the standard 0.05 (raw *p*-value/5).

## Results

### Clinical characteristics of patients

Of the 192 HIV/CMV-coinfected patients tested using a commercial polymerase chain reaction (PCR) assay, 161 (83.9%) had undetectable levels of HIV RNA. The CMV IgG antibodies were detected in all patients with HIV/CMV coinfection. Forty-one subjects had a CD4^+^ T cell count ≤ 200 cells/mm^3^ (including 28 patients with CMV DNAemia and 13 subjects without CMV DNAemia), and 151 had a CD4^+^ T cell count > 200 cells/mm^3^ (including 80 patients with CMV DNAemia and 71 subjects without CMV DNAemia; Table 1).

### The heterozygous genotype of the *TLR3* rs3775291 SNP is prevalent in patients with CMV DNAemia

First, the *DDX58* rs10813831, rs12006123; *IFIH1* rs1990760, rs3747517; and *TLR3* rs3775291 and rs3775296 SNPs were genotyped in 192 HIV/CMV-coinfected patients. The *DDX58* rs12006123 GG genotype and the *TLR3* rs3775296 CC genotype were detected less frequently in HIV/CMV-coinfected patients with CMV DNAemia than in subjects without CMV DNAemia (22.2% *vs.* 41.7%, *p* = 0.004, and 28.7% *vs.* 46.4%, *p* = 0.011, respectively). The heterozygous (CT) genotype of the *TLR3* rs3775291 polymorphism was more common in HIV/CMV-coinfected patients with CMV DNAemia than in those without CMV DNAemia (56.5% *vs.* 39.3%, *p* = 0.018). No significant differences were found for the *DDX58* rs10813831 and *IFIH1* rs1990760 and rs3747517 SNPs. Interestingly, the homozygous recessive (AA) genotype of the *DDX58* rs12006123 SNP was observed more frequently in HIV/CMV-coinfected patients with CD4^+^ T counts below 200 cells/mm^3^ than in those with CD4^+^ T counts > 200 cells/mm^3^ (OR, 4.60; 95% CI, 1.61–13.13; *p* = 0.013). A higher prevalence of low CD4^+^ T-cell counts (CD4^+^ T-cell count ≤ 200 cells/mm^3^) in HIV/CMV-coinfected patients with a heterozygous (GA) or homozygous recessive (AA) genotype of the *DDX58* rs10813831 polymorphism (OR, 2.62; 95% CI, 1.27–5.40; *p* = 0.007) was also noted. The observed genotype and allele frequencies of the *TLR3* rs3775296 SNP were not in HWE (*p* > 0.05) and were excluded from further analysis.

### The *DDX58* rs12006123 SNP is frequent in HIV/CMV-coinfected patients with CMV DNAemia

The heterozygous (GA) and homozygous recessive (AA) genotypes of *DDX58* rs12006123 occurred more frequently in HIV/CMV-coinfected patients with CMV DNAemia than in coinfected subjects without CMV DNAemia (OR, 2.50; 95% CI, 1.33–4.68; *p* = 0.004, in the dominant model; Table 2). This SNP showed a higher rate of occurrence of CMV DNAemia even after Bonferroni correction for multiple tests (*p*_*B*_ = 0.010). The heterozygous (CT) genotype of the *TLR3* rs3775291 SNP was detected more commonly in HIV/CMV-coinfected patients with CMV DNAemia than in those without CMV DNAemia (OR, 2.01; 95% CI, 1.12–3.58; *p* = 0.018, in the overdominant model). However, this finding did not reach statistical significance after Bonferroni correction for multiple tests (*p*_*B*_ > 0.010).

**Table 2:** Association between SNPs in PRR genes and CMV DNAemia among HIV/CMV-coinfected patients

*PRR* SNP	Model	Genotype	Genotype frequencies; *n* (%)^a^		Unadjusted		Adjusted^b^		
			Patients with CMV DNAemia	Patients without CMV DNAemia	OR (95% CI)	*p*-value	OR (95% CI)	*p*-value	
*DDX58*									
rs10813831	Codominant	GG	51 (47.2)	50 (59.5)	1.00	0.12	1.00	0.068	
		GA	49 (45.4)	26 (31.0)	1.85 (1.00-3.42)		2.15 (1.10–4.20)		
		AA	8 (7.4)	8 (9.5)	0.98 (0.34–2.82)		1.07 (0.34–3.37)		
	Dominant	GG	51 (47.2)	50 (59.5)	1.00	0.09	1.00	0.044	
		GA-AA	57 (52.8)	34 (40.5)	1.64 (0.92–2.93)		1.90 (1.01–3.56)		
	Recessive	GG-GA	100 (92.6)	76 (90.5)	1.00	0.6	1.00	0.64	
		AA	8 (7.4)	8 (9.5)	0.76 (0.27–2.12)		0.76 (0.25–2.32)		
	Overdominant	GG-AA	59 (54.6)	58 (69.0)	1.00	0.041	1.00	0.02	
		GA	49 (45.4)	26 (31.0)	1.85 (1.02–3.37)		2.13 (1.11–4.07)		
rs12006123	Codominant	GG	24 (22.2)	35 (41.7)	1.00	0.009	1.00	0.025	
		GA	60 (55.6)	39 (46.4)	2.24 (1.16–4.33)		2.27 (1.12–4.60)		
		AA	24 (22.2)	10 (11.9)	3.50 (1.42–8.63)		3.09 (1.18–8.10)		
	Dominant	GG	24 (22.2)	35 (41.7)	1.00	0.004	1.00	0.009	
		GA-AA	84 (77.8)	49 (58.3)	2.50 (1.33–4.68)		2.44 (1.24–4.80)		
	Recessive	GG-GA	84 (77.8)	74 (88.1)	1.00	0.059	1.00	0.15	
		AA	24 (22.2)	10 (11.9)	2.11 (0.95–4.71)		1.85 (0.79–4.34)		
	Overdominant	GG-AA	48 (44.4)	45 (53.6)	1.00	0.21	1.00	0.17	
		GA	60 (55.6)	39 (46.4)	1.44 (0.81–2.56)		1.53 (0.82–2.85)		
*IFIH1*									
rs1990760	Codominant	CC	36 (33.3)	34 (40.5)	1.00	0.56	1.00	0.84	
		CT	58 (53.7)	39 (46.4)	1.40 (0.76–2.61)		1.21 (0.62–2.35)		
		TT	14 (13.0)	11 (13.1)	1.20 (0.48–3.01)		1.20 (0.46–3.13)		
	Dominant	CC	36 (33.3)	34 (40.5)	1.00	0.31	1.00	0.87	
		CT-TT	72 (66.7)	50 (59.5)	1.36 (0.75–2.46)		1.07 (0.44–2.61)		
	Recessive	CC-CT	94 (87.0)	73 (86.9)	1.00	0.98	1.00	0.65	
		TT	14 (13.0)	11 (13.1)	0.99 (0.42–2.31)		1.15 (0.63–2.13)		
	Overdominant	CC-TT	50 (46.3)	45 (53.6)	1.00	0.32	1.00	0.49	
		CT	58 (53.7)	39 (46.4)	1.34 (0.76–2.37)		1.24 (0.68–2.26)		
rs3747517	Codominant	TT	11 (10.2)	4 (4.8)	1.00	0.33	1.00	0.33	
		TC	43 (39.8)	33 (39.2)	1.13 (0.62–2.06)		1.08 (0.57–2.06)		
		CC	54 (50.0)	47 (56.0)	2.39 (0.71–8.02)		2.51 (0.71–8.92)		
	Dominant	TT	11 (10.2)	4 (4.8)	1.00	0.15	1.00	0.5	
		TC-CC	97 (89.8)	80 (95.2)	2.27 (0.70–7.40)		1.24 (0.67–2.29)		
	Recessive	TT-TC	54 (50.0)	37 (44.0)	1.00	0.41	1.00	0.14	
		CC	54 (50.0)	47 (56.0)	1.27 (0.72–2.25)		2.43 (0.71–8.37)		
	Overdominant	TT-CC	65 (60.2)	51 (60.8)	1.00	0.94	1.00	0.91	
		TC	43 (39.8)	33 (39.2)	1.02 (0.57–1.83)		0.97 (0.52–1.81)		
*TLR3*									
rs3775291	Codominant	CC	22 (20.4)	27 (32.1)	1.00	0.05	1.00	0.044	
		CT	61 (56.5)	33 (39.3)	2.27 (1.12–4.59)		2.48 (1.15–5.36)		
		TT	25 (23.1)	24 (28.6)	1.28 (0.58–2.83)		1.34 (0.56–3.18)		
	Dominant	CC	22 (20.4)	27 (32.1)	1.00	0.064	1.00	0.056	
		CT-TT	86 (79.6)	57 (67.9)	1.85 (0.96–3.56)		2.00 (0.98–4.11)		
	Recessive	CC-CT	83 (76.9)	60 (71.4)	1.00	0.39	1.00	0.38	
		TT	25 (23.1)	24 (28.6)	0.75 (0.39–1.44)		0.73 (0.36–1.47)		
	Overdominant	CC-TT	47 (43.5)	51 (60.7)	1.00	0.018	1.00	0.016	
		CT	61 (56.5)	33 (39.3)	2.01 (1.12–3.58)		2.14 (1.15–3.99)		

^a^ Values are the number of subjects examined (%);

^b^ Adjusted analysis was carried out for age, sex, HIV RNA load, and CD4^+^ T cell count in blood samples; OR, odds ratio; 95% CI, 95% confidence interval; *p*, logistic regression model. The *p*-value after Bonferroni correction for multiple testing is 0.010 (raw *p*-value/5).

### PRR SNPs are associated with CMV DNAemia

Of the 192 HIV/CMV-coinfected patients, 56.3% had detectable levels of CMV DNA in their peripheral blood. A higher level of CMV viremia was observed in patients who were heterozygous (GA) or homozygous recessive (AA) for the *DDX58* rs12006123 SNP (median, 1.31 × 10^2^ copies/ml; range, 0-3.22 × 10^6^ copies/ml) than in those who had a wild-type (GG) genotype for this polymorphism (median, 9.40 × 10^2^ copies/ml; range, 0-1.02 × 10^4^ copies/ml; *p* = 0.0003). The median CMV DNAemia was also higher among subjects who were homozygous recessive (TT) for the *TLR3* rs3775291 SNP (median, 2.78 × 10^2^ copies/ml; range, 0-3.22 × 10^6^ copies/ml) compared with those who were heterozygous (CT) or had a wild-type (CC) genotype for this polymorphism (median, 1.36 × 10^2^ copies/ml; range, 0-9.34 × 10^5^ copies/ml; *p* = 0.047). No other associations were found (*p* > 0.05).

### Haplotype analysis

Haplotype analysis of *DDX58* rs10813831, rs12006123; *IFIH1* rs1990760, rs3747517; and *TLR3* rs3775291 SNPs revealed two haploblocks: *DDX58* and *IFIH1*. The haplotype GA of the *DDX58* haploblock was detected in 37.0% of HIV/CMV-coinfected patients with CMV DNAemia and in 25.5% of coinfected subjects without CMV DNAemia and was associated with a twofold increased incidence of CMV DNAemia (OR, 2.10; 95% CI, 1.21–3.37; *p* = 0.010). For the *IFIH1* haploblock, the most prevalent haplotype was CC (59.3% of cases); however, no association with the incidence of CMV DNAemia in HIV/CMV-coinfected patients was noted. A tight LD was found for the adjacent SNPs in the coding region of *IFIH1* rs3747517 and rs1990760 (r^2^ = 0.45).

### Association of PRR SNPs with cytokine production

To investigate the clinical relevance of PRR SNPs and the outcome of coinfection, the serum concentrations of 10 target cytokines in the examined patients were measured (Table 1). The levels of IL-2, IL-17A, and IFN-γ were below the standard range in all patients. In subjects with a mutation detected in at least one allele of the *DDX58* rs12006123 SNP, a lower serum IFN-β concentration was found compared with those who had a wild-type (GG)

genotype for this polymorphism (median, 0 pg/ml; range, 0-2935 pg/ml *vs.* median, 209.7

pg/ml; range, 0-574.7 pg/ml; *p* = 0.024). Individuals with a wild-type (CC) genotype for the *TLR3* rs3775291 SNP had a higher serum IFN-β concentration than those who were heterozygous (CT) or homozygous recessive (TT) for this polymorphism (median, 0 pg/ml; range, 0-2935 pg/ml *vs.* median, 0 pg/ml; range, 0-2922 pg/ml; *p* = 0.026). Moreover, the serum IL-7 concentration was lower in HIV/CMV-coinfected patients with the *TLR3* rs3775291 CC and CT genotypes compared with patients with the TT genotype (median, 37.3 pg/ml; range, 0-1100.0 pg/ml *vs.* median, 55.0 pg/ml; range, 4.4-825.1 pg/ml; *p* = 0.041). No other cytokines showed associations with PRR SNPs.

## Discussion

To our knowledge, this is the first study to assess the relationship between TLR3 and RLR gene polymorphisms and the occurrence of CMV DNAemia in HIV/CMV-coinfected adults. The *DDX58* rs12006123 and *TLR3* rs3775291 SNPs were associated with a higher incidence of CMV DNAemia among HIV/CMV-coinfected patients as well as with higher DNA CMV loads in the peripheral blood. Mutations present in at least one allele of these polymorphisms were associated with a lower serum IFN-β concentration, and a link between the CD4^+^ T cell count and PRR SNPs was noted.

HIV-1, a member of the family *Retroviridae*, is one of the most intriguing and challenging viruses of the last century. The present study revealed that the *DDX58* rs12006123 and the *TLR3* rs3775291 SNPs are associated with a higher incidence of CMV DNAemia in HIV/CMV-coinfected patients and with higher DNA CMV loads. It was reported recently that the *TLR3* rs3775291 CT genotype was associated with the early stage of HIV infection among individuals naïve to ART, with a higher frequency in the advanced stage of HIV infection compared to healthy subjects [13]. Interestingly, the heterozygous (GA) genotypes of the *DDX58* rs12006123 and rs10813831 SNPs were more frequently observed in HIV/CMV-coinfected individuals with CD4^+^ T counts below 200 cells/mm^3^. It is well known that the persistence of latent HIV proviruses in CD4^+^ T cells, despite combined ART, is a major roadblock to HIV eradication. It was found recently that stimulation of the RIG-I pathway enhanced RIG-I signaling *ex vivo*, increased HIV transcription, and induced apoptosis of HIV-infected CD4^+^ T cells [14]. Other studies, however, showed that RIG-I-dependent IFN activation was not significant and was insufficient to induce the death of latently infected HIV-positive cells [15, 16]. We therefore hypothesize that cells carrying *DDX58* polymorphisms might act as a cellular reservoir of HIV that takes advantage of the immune environment to facilitate HIV persistence and replication.

PRRs are a family of germline-encoded receptors that play a pivotal role in the host’s early response to invading pathogens and subsequent adaptive immunity. TLR3 and RIG-I recognize double-stranded RNA (dsRNA), produced during viral replication or release from apoptotic cells [15, 17, 18], and activate specific signaling pathways that lead to induction of type I and III IFN and/or production of inflammatory cytokines. However, the mechanism by which *DDX58* rs12006123 polymorphisms regulate the host response against HIV/CMV coinfection remains unclear. The *DDX58* rs12006123 SNP is located in the 3’UTR region of the RIG-I gene and does not lead to an amino acid change. It was reported that the *DDX58* rs12006123 SNP did not affect allele-specific mRNA expression in human dendritic cells [11]. However, one study has shown a significant reduction in measles-virus-induced IFN-γ and IL-2 secretion in peripheral blood mononuclear cells of measles-virus-infected patients with the *DDX58* rs12006123 SNP [19].

In the present study, the *TLR3* rs3775291 CC and CT genotypes were associated with a lower serum IL-7 concentration. IL-7 is known to be essential for *de novo* T cell generation in the thymus, and it contributes to the maintenance of peripheral T cell homeostasis. It has been shown that the CD4^+^ and CD8^+^ T cell populations downregulate the IL-7 receptor in response to IL-7 and other pro-survival cytokines (e.g., IL-2, IL-4, IL-6, and IL-15), and IL-7 also contributes to T cell development, homeostatic proliferation, and survival [20, 21]. Recently, clinical trials have shown that repeated cycles of recombinant IL-7 injections are safe and could improve the immune response by affecting CD4^+^ T cell proliferation and survival [22, 23]. It is therefore plausible that a cellular mechanism (e.g., immune reconstitution inflammatory syndrome) or other SNPs in linkage disequilibrium can have a direct effect on the individual SNP.

There are some potential limitations to this study. First, this work was conducted on a small number of HIV/CMV-coinfected volunteers. Second, all HIV/CMV-coinfected subjects received combined ART therapy, which can modulate the immune response. Therefore, larger studies are necessary in the future to confirm the association between PRR polymorphisms and CMV DNAemia in HIV-infected subjects.

Overall, this study showed that the *DDX58* rs12006123 polymorphism might be associated with a higher incidence of CMV DNAemia in HIV/CMV-coinfected adults. An effect of PRR polymorphisms on cytokine concentrations was also observed. The results suggest a broad area for further studies of the dynamic nature of the intermolecular interactions between pathogens and host receptors.

## Data Availability

The current study has no associated data.
